# Influence of Cement Replacement with Sewage Sludge Ash (SSA) on the Heat of Hydration of Cement Mortar

**DOI:** 10.3390/ma15041547

**Published:** 2022-02-18

**Authors:** Elżbieta Haustein, Aleksandra Kuryłowicz-Cudowska, Aneta Łuczkiewicz, Sylwia Fudala-Książek, Bartłomiej Michał Cieślik

**Affiliations:** 1 Department of Mechanics of Materials and Structures, Faculty of Civil and Environmental Engineering, Gdańsk University of Technology, Narutowicza 11/12, 80-233 Gdańsk, Poland; 2 Department of Water and Wastewater Technology, Faculty of Civil and Environmental Engineering, Gdańsk University of Technology, Narutowicza 11/12, 80-233 Gdańsk, Poland; aneta.luczkiewicz@pg.edu.pl; 3 Department of Sanitary Engineering, Faculty of Civil and Environmental Engineering, Gdańsk University of Technology, Narutowicza 11/12, 80-233 Gdańsk, Poland; sksiazek@pg.edu.pl; 4 Department of Analytical Chemistry, Faculty of Chemistry, Gdańsk University of Technology, Narutowicza 11/12, 80-233 Gdańsk, Poland; bartlomiej.cieslik@pg.edu.pl

**Keywords:** sewage sludge ash, cement mortar, hydration heat evolution, isothermal calorimetry

## Abstract

The amount of fly ash from the incineration of sewage sludge is increasing all over the world, and its utilization is becoming a serious environmental problem. In the study, a type of sewage sludge ash (SSA) collected directly from the municipal sewage treatment plant was used. Five levels of cement replacement (2.5%, 5%, 7.5%, 10% and 20%) and unchanged water-to-binder (w/b) ratio (0.55) were used. The purpose of the study was to evaluate the effect of sewage sludge ash (SSA) on the hydration heat process of cement mortars. The heat of the hydration of cement mortars was monitored by the isothermal calorimetric method for 7 days at 23 °C. The analysis of chemical composition and particle size distribution was performed on the tested material. The tests carried out have shown that SSA particles have irregular grain morphology and, taking into account the chemical composition consists mainly of oxides such as CaO, P_2_O_5_, SiO_2_ and Al_2_O_3_. The concentration of these compounds affects the hydration process of cement mortars doped with SSA. In turn, the content of selected heavy metals in the tested ash should not pose a threat to the environment. Calorimetric studies proved that the hydration process is influenced by the presence of SSA in cement mortars. The studies showed that the rate of heat generation decreased (especially in the initial setting period) with the increasing replacement of cement by SSA, which also reduced the amount of total heat compared to the control cement mortar. With increasing mass of the replacement of cement with SSA up to 20%, the 7-day compressive strength of the mortar samples decreases.

## 1. Introduction

Sewage sludge ash (SSA) is the by-product generated during the incineration of dewatered sewage sludge. Fluidization bed combustion (FBC) is one of the widely used thermal utilization technologies. Combustion-based methods implemented for sewage sludge utilization result in the production of significant amounts of fly ash, classified by EU/2014/955 [1] as waste coded 19 01 14, which should be properly recycled. Coal-fired thermal power plants also produce large amounts of combustion waste, such as fly ash, solid residues from exhaust gas treatment systems or slags [2]. Landfilling or reclamation of degraded areas are commonly used for further management of SSA; however, both methods may pose a direct threat to the environment, by simultaneous earth, air and water pollution. Thus, the possibility of using SSA in the construction industry is widely discussed, as a step toward a circular economy and an alternative way to develop building materials, meeting the growing demand in the construction industry. Researchers are constantly looking for modern solutions affecting the reduction of energy consumption and CO_2_ emissions [3,4]. An option worth considering is to use SSA in cementitious materials as a cement substitute. It is important to know that the utilization of SSA as a supplementary cementitious material (SCM) to replace cement or fine aggregate (sand) in concrete introduces several economic and technical benefits, such as indirect protection of natural resources. On the other hand, discussed material have not yet been fully accepted as a mineral additive to cement or concrete.

According to Yusuf et al. [5] and Vouk et al. [6] the main elements present in SSA are Al, Ca, Fe, P and Si. However, others also indicate oxygen, since most of the mentioned elements form oxides (Al_2_O_3_, CaO, Fe_2_O_3_, P_2_O_5_, or SiO_2_) during the thermal utilization process [7]. It is also important to note that there may also be significant amounts of as SO_3_ surface adsorbed species. The reoccurrence of mentioned elements and some chemical compounds is usually a result of certain technological processes used in wastewater treatment plants (e.g., phosphorus precipitation by Al/Fe salts, mainly sulfates and chlorides) and the thermal utilization technology by itself. Therefore, the main crystalline phases in SSA are silicon oxide (SiO_2_), calcium phosphate (Ca_3_(PO_4_)_2_) and hematite (Fe_2_O_3_). Among them, phosphates are suggested to delay the setting time and generally reduce the initial concrete strength [5,6]. Vouk et al. [6] concluded that the effect of SSA on the setting time is determined by the chemical composition. CaO, MgO and chlorides accelerate the binding sulphate effect on the setting time, and strength development, while the phosphate content (in the form of P_2_O_5_) affects the final setting time in concentrations higher than 0.3%.

Several authors have studied the effect of the addition of SSA on the leaching of heavy metals from cementitious materials on the environment. Chen et al. [8] and Donatello et al. [9] concluded that the leaching level was within the allowable limits according to the EN 15,863 standard [10]. According to Coutand et al. [11], the degree of leaching of heavy metals from cement mortars with SSA was of the same order of magnitude as that of reference mortars without SSA. Cenni et al. [12] analyzed the leaching of heavy metals such as Cd, Cu, Cr, Ni, Pb and Zn from ash derived from coal and sewage sludge co-firing and concluded that the concentration of these metals in the extracts was below the detection limit. The results presented by Latosińska et at. [13] pointed out that the addition of SSA improved the strength parameters and frost resistance of concrete and also met the environmental requirements for heavy metal leaching. Wichowski et al. [14] concluded that the migration of heavy metals from concrete with the participation of SSA was negligible and probably does not pose a threat to the environment.

The particle size distribution of SSA ranges from 1 to 100 µm, with an average diameter of about 26 µm. Its specific density is usually in the range of 2.3–3.2 g/cm^3^ [7,15]. In turn, Vouk et al. [15] suggest that the density of SSA is influenced by the combustion temperature of the sewage sludge. They found that the densities of the SSA increase with increasing temperature from 2.67 g/cm^3^ at 800 °C to 2.83 g/cm^3^ at 1000 °C.

The specific surface areas of SSA (according to Blaine and the Brunnauer, Emmett and Teller (BET)) are 640 and 19,000 m^2^/kg, respectively. The latter value, which is quite high, might be related to the morphological irregularities of the grains, as suggested by Monzó et al. [16], Coutand et al. [11], Baeza-Brotons et al. [17] and Vouk et al. [6]. Jamshidi et al. [18] determined the pozzolanic activity of SSA at 37.9% after 28 days, while Fontes et al. [19] proposed a value of 70.5%.

Many researchers (Monzó et al. [20], Cyr et al. [21], Lin et al. [22], Garcés et al. [23], Cusidó et al. [24], Agrawal et al. [25], Baeza-Brotons et al. [17], Lynn et al. [26]) have suggested that SSA may be used as an addition in the production of building materials such as mortar, concrete, brick, asphalt paving mixes, aerated concrete, lightweight and heat-insulating material, ceramic tiles, eco-cement and for soil stabilization. However, preserving mineral resources by replacing them with reused/recycled waste needs to be evaluated in a holistic way, combining the practical assay with economic prosperity and human well-being with a healthy environment. In the case of SSA, it was stated that the origin of wastewater, the type of treatment used, and the sewage sludge incineration system may cause changes in the properties of concrete and composite materials [27]. Lin and Lin [28], Yusuf et al. [5], Monzó et al. [29], Chin et al. [30] and Ingunza et al. [31] stated that SSA can be used as a substitute for cement in mortars and concrete. The researchers concluded that SSA could replace part of the cement in the mortar, as long as this level of replacement is below 20%. In turn, Pan et al. [32], Donatello et al. [33] and Kappel et al. [34] investigated the effect of SSA with different degrees of fineness on the properties of final cement mortars. They established that increasing the degree of fineness of SSA improves the mechanical features (strength) and workability of mortars. However, there are many authors who claim the opposite [7]. Piasta and Lukawska [35] suggested that the strength gain for mortars containing 10% and 20% SSA was slower than for mortar with Portland cement only. The mentioned researchers found that the high content of phosphorus oxide in SSA increases the final setting time.

The main hydration products in pure cement are C-S-H gel, Ca(OH)_2_ and unhydrated C_3_S and C_2_S. Investigations of SSA cement pastes using the X-ray diffraction analysis (XRD) method (Lin and Lin [28]) showed that the intensity peaks of SSA cement pastes were basically the same as the normal Portland cement pastes (OPC), while the C_3_S and C_2_S diffraction peaks clearly decreased after 28 days.

According to Dyer et al. [36] the heat of hydration of SSA and Portland cement mixtures requires detailed research, because the waste is often rich in phosphates, distinguishing it from other mineral additives used in concrete technology. Monzó et al. [16] examined the evaluation of SSA reactivity and showed that CH consumption decreased from 29% to 16% in the first three days of curing, and the observed increase in temperature tended to activate the pozzolanic reaction. Dyer et al. [37] used an isothermal conduction calorimeter and XRD to assess the hydration of cement pastes containing SSA and observed the presence of hydrated carboaluminates and aluminosilicates. They also detected other minerals, such as monosulfate.

Despite numerous studies of the ash from sewage sludge as a component of cement or concrete, there is still little information on the effect of its amount on the hydration heat of the cement matrix. Isothermal calorimetry is a non-destructive and non-invasive method used for estimating the heat of hydration of building materials, which is the main advantage of the applied method.

The aim of this study was to evaluate the trend of changes in the hydration process of SSA, which was introduced into mortars as a cement replacement. A series of isothermal calorimetric tests (at 23 °C) with different SSA content were performed to evaluate how SSA affects hydration heat within seven days. The studies were carried out on the SSA obtained from fluidized combustion processes, collected from one municipal wastewater treatment plant located in Poland.

## 2. Materials and Methods

The following materials were used to prepare the cement mortar mixtures: ordinary Portland cement (OPC, class 42.5 R, Cement Company “Górażdże”, Poland) according to European Standard EN 197-1 [38], natural sand (Aggregate Exploitation Plant “Borowiec”, Gdańsk-Chwaszczyno, Poland) with maximum grain size of up to 2 mm and tap water. The main research material was fly ash (classified according to the European Waste Catalogue [1] as code 19 01 14) formed in a thermal utilization facility in which sewage sludge is burned in a fluidized bed furnace at a temperature above 850 °C. The SSA samples were collected from one municipal wastewater treatment plant located in Poland: Sewage Sludge Treatment Plant “Wschód” in Gdańsk.

In the present work, five substitution rates of cement to SSA were used—2.5, 5, 7.5, 10 and 20%. In all cases, the water/binder ratio of the mortar was 0.55. In order to compare the effect of SSA addition, a reference mixture (control test) without SSA was made. The composition of cement mortars without and with SSA is presented in Table 1.

The main SSA features identified in the present studies concerned the determination of their chemical composition including selected heavy metals, specific density and particle size distribution in the tested material.

The morphology and chemical composition were observed with the use of scanning electron microscope (SEM, type JEOL JSM 7800 F, Akishima, Tokyo, Japan) equipped with energy dispersive X-ray detector—EDAX (Octane Elite, Mahwah, NJ, USA). The loss on ignition (LOI) was identified according to EN 196-2 [39]. The application of this technique allows us to determine the chemical composition of cementitious materials and the microstructure of concrete [40,41,42].

The content of heavy metals, such as Cd, Cu, Ni, Pb, Zn and Cr, in the eluate from SSA sample was determined by atomic absorption spectrometry (AAS) using a Vario 6 Spectrometer (Analytik Jena AG, Jena, Germany) with air-acetylene flame.

The particle size distribution (PSD) of the raw materials used in this study (OPC and SSA) were analyzed with a laser particle analyzer (Helios/R, Sympatec GMbH, Clausthal-Zellerfeld, Germany). According to the International Standard ISO 13,320 [43] the method mentioned is a standardized procedure used for the determination of the particle size distribution. The analyzer applies to the rapid and automatic particle size analysis of solids by so called dry method. The range of operation of the analyzer is from 0.1 to 3500 μm. The volume particle size distribution (PSD) was described by D-values (D_10_, D_50_ and D_90_), which identify the 10th, 50th and 90th percentiles below a given particle diameter than the calculated diameter based on the results from the laser analysis. D_50_ is also defined as the median size; that is, the size that splits the size distribution with half above and half below the specified diameter. The mean size or mean particle size (VMD) expresses the volume mean as an average of D_10_, D_50_ and D_90_.

The specific density of the OPC and SSA were determined in accordance with ASTM C188 [44]. The tests were carried out using liquid naphtha with a density greater than 0.73 g/mL at 23 °C.

The study of fineness, i.e., residue on a 0.045 mm sieve of the raw materials used in this study (OPC and fly ash sample) were carried out using the wet method in accordance with the requirements of EN 451-2 [45].

The heat parameters of the cement mortars were determined using the 3-channel TAM Air isothermal conduction calorimeter (ICC). ICC tests were performed in accordance with the recommendations described in the ASTM C1679 standard [46]. The hydration process was represented by the hydration heat evolution curves for mortars with different SSA substitution rates. Kuryłowicz-Cudowska et al. [47,48] proved that monitoring thermal parameters is also useful for the estimation of the strength of cementitious materials.

**Figure 1:**
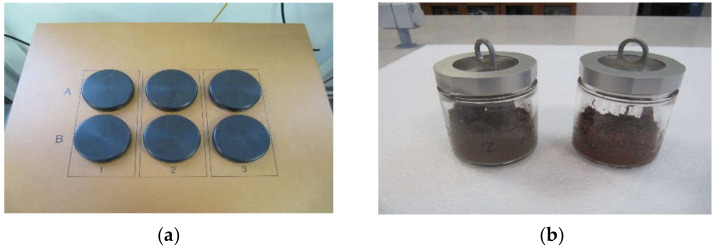
(**a**) Profiles of 3-channel TAM Air; (**b**) mortar specimens in a glass ampule.

The isothermal calorimeter was used to measure the degree of reaction of the SSA. This was done by replacing the cement in the cement mortar with SSA, to determine the effect on the heat of hydration. The study of quartz-based and ash-based mortar tests allows a comparison of the hydration process without and with the tested materials [49]. The TAM Air calorimeter used in study has double channels for test and reference samples, each with a volume of 125 mL. As shown in Figure 1a, the channel marked “A” was used for cement mortar samples, while the channel marked “B” was used for the reference samples. The reference samples were prepared by substituting the cement mass with quartz sand.

The thermostat uses circulating air with an advanced temperature regulating system to maintain a highly stable temperature (within ±0.02 °C). The temperature change between the sample and the surroundings (maintained at constant temperature) results in heat flow. Heat changes were continuously monitored. The high accuracy and stability of the thermostat make the calorimeter well suited for measurements of heat flow over long periods [50].

The cement mortars (Table 1), were carried out manually by mixing cement, quartz sand, SSA and tap water together with a glass rod in a glass calorimetric ampule (Figure 1b). The closed ampoule was placed in a calorimeter to monitor heat evolution for 7 days.

Cumulative hydration heat *Q* and heat flow dQ/dt were measured at 23 °C, which was the temperature in the laboratory. Isothermal conditions mean maintaining a constant temperature throughout the test. During each test, the data was continuously recorded for 7 days (168 h) using a three-channel data logger connected to the computer. The baseline was measured for a minimum of 24 h to achieve signal stability conditions using the linear least-squares procedure (absolute value of slope less than 3 μW/h and standard deviation less than 12 μW).

Strength activity index (SAI) according to EN 451-1 [51] determines the pozzolanic activity of SSA. SAI is an indirect method to evaluate the activity of the study material by determining the compressive strength of the test mortar. According to EN 450-1 [51], the active strength index of SSA was tested by replacing 25% of cement with ash from sewage sludge. The test results are presented in Section 3.3.

Compressive strength tests were carried out in order to compare the obtained results with the heat of hydration of the mortar samples. The tests were performed using 50 mm mortar cubes in accordance with ASTM C109/C109M [52]. The samples were cured in water bath at a temperature corresponding to the heat of hydration test temperature (23 °C). The test results at 1, 3, 5 and 7 days are presented in Section 3.4.

The mineralogical composition of mortar samples at 7 days was determined using an X-ray diffractometer (XRD, MiniFlex 600, Rigaku Co., Tokyo, Japan). The testes were carried performed with a copper tube, radiation generated at 20 mA and 40 KV, in the angular range from 5 to 90°, with a scanning rate of 5°⋅min^−1^. The raw XRD data obtained from the spectrometer were analyzed by PDXL software. The test results are presented in Section 3.6 [42].

## 3. Results and Discussion

### 3.1. The Chemical Composition and the Physical Properties of the Raw Materials (OPC, SSA)

**Figure 2:**
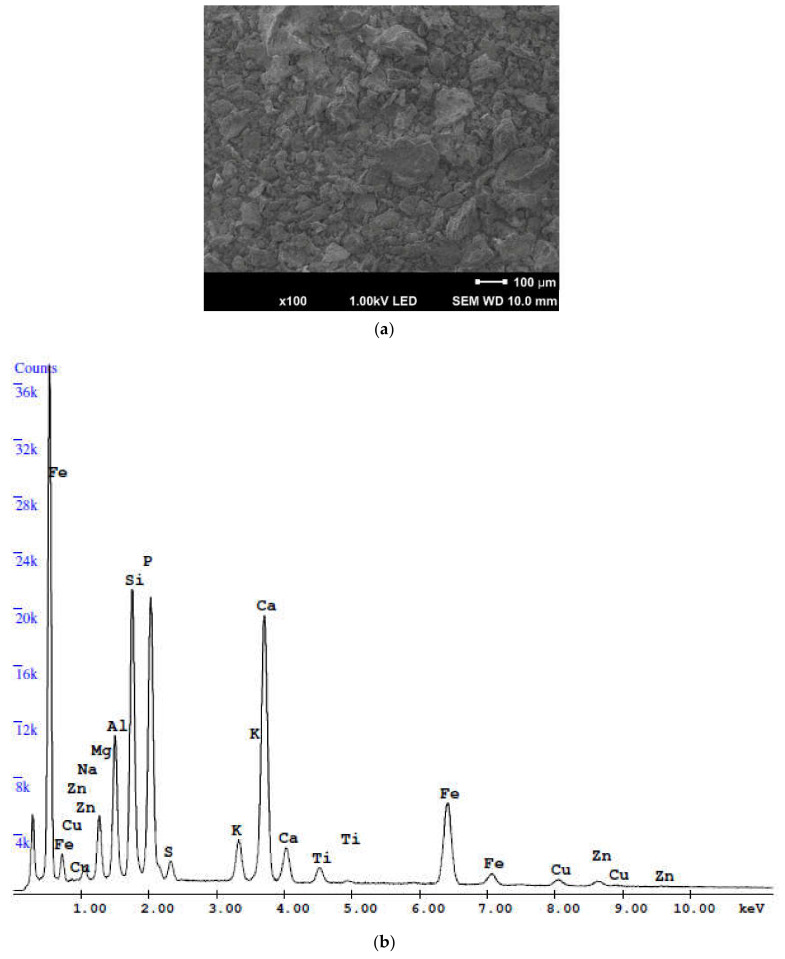
(**a**) Scanning electron microscopy (SEM) image, magnification 100×; (**b**) energy dispersive X-ray spectrometer (EDS) analysis of the sewage sludge ash (SSA) sample after thermal incineration of sewage sludge.

The results of the SEM analysis (Figure 2a) show that the SSA particles are a collection of irregular grains, which in most cases seem to be an aggregation of small particles. Their rough surface textures can lead to high water absorption of ash-enriched cement mortars. This is confirmed by the studies of Monzó et al. [29], suggesting that the irregular morphology of SSA particles reduces workability and changes the consistency of cement mortar or concrete.

The chemical compositions and loss on ignition (LOI) of the raw materials (OPC and SSA) used in this study are presented in Table 2, and compared with the requirements of the EN 450-1 standard [51]. The concentrations of selected heavy metals (mg/kg dry weight) of the ash sample (SSA) are shown in Table 3, respectively.

The chemical composition of the ash produced in the combustion process performed in a fluidized bed furnace is a derivative of the chemical composition of the wastewater supplied to the treatment plant and the treatment technology used. Depending on the quality of sewage sludge and the incineration parameters, fly ash generated during the thermal utilization process tends to differ significantly in chemical composition and physical properties (Table 2).

Ordinary Portland cement (OPC, class 42.5 R) used in the presented study is a powdery substance made of clinker and gypsum. Taking into account the content, the main oxides in cement are CaO (64.1%) and SiO_2_ (21.2), while the minor oxides include Al_2_O_3_ (5.0%), SO_3_ (3.0%), MgO (2.5%), Fe_2_O_3_ (2.2%) and some alkali oxides (K_2_O and Na_2_O).

The data presented in Table 2 show that samples (SSA) contain mainly phosphorus pentoxide (P_2_O_5_), silicon dioxide (SiO_2_) and calcium oxide (CaO). The other oxides, such as MgO, SO_3_, Na_2_O and K_2_O, are present in smaller amounts.

The content of silica oxide (SiO_2_) in the ash sample (SSA) is less than 25% by weight, while aluminum oxide (Al_2_O_3_) is 9.6%, respectively. According to Yu et al. [53], silica is one of the most important chemical compounds in concrete, required in the pozzolanic reaction to produce calcium-silica-hydroxide (C-S-H phase). Therefore, it is usually suggested that SSA may adversely affect the durability and strength of concrete. The iron oxide content (Fe_2_O_3_) is six times higher in the SSA sample compared to OPC (Table 2). It is associated with the use of Fe-based (or Al-based) salts for precipitation of phosphorus from wastewater during the wastewater treatment process. SSA has a characteristic red iron oxide color, which is the reason for the change in cement mortar color from light red to dark red.

The amount of calcium oxide (CaO) is 18.2% for SSA (Table 2). Calcium oxide has an important function during the cement hydration process; mainly because it affects the formation of calcium silicates and aluminate in the structure of concrete.

The chemical composition (Table 2) indicates that the sulfur trioxide (SO_3_) content in cement (3.0%) is higher than in the SSA sample, where the amount of this oxide is equal to 2.3% of the total weight. Many studies have proven that the sulfates contained in Portland cement control C_3_A hydration, but the total amount should not be too high to avoid undesirable swelling (in early and late age) of the concrete. According to Coutand et al. [11], the sulfates should be highly soluble; otherwise, late dissolution could lead to the formation of delayed ettringite.

The concentration of alkalis expressed as sodium equivalent Na_2_O_eq_ (= Na_2_O + 0.658K_2_O) is 2.6% in SSA, whereas in ordinary Portland cement (OPC) equals only 0.74%.

The phosphorus pentoxide (P_2_O_5_) in the sample is about 25.0% (Table 2). In this case, the presence of phosphorus pentoxide in SSA is caused mainly by the removal of soluble P in activated sludge processes (by phosphate accumulating organisms), occasionally supported by precipitation with abovementioned Fe- or Al-based precipitating agents.

According to Latosińska et al. [13] the phosphate ions hinder the cement hydration process, extending the beginning of its binding time. Under the influence of water, PO_4_^3−^ ions react with Ca^2+^ ions in the liquid phase of the binder, resulting in precipitation on the surface of the cement grains of fine, hard-soluble calcium phosphate Ca_3_(PO_4_)_2_ that interferes with the rate of nucleation and crystal growth, which are the hydration products of Portland clinker.

According to the data presented in Table 2, the total content of three basic oxides (SiO_2_ + Al_2_O_3_ + Fe_2_O_3_) in SSA is 42.3% by weight. The values obtained do not meet the requirements of two standards: EN 450-1 [51] and ASTM C618 [54], in relation to fly ash generated during coal combustion or co-combustion of coal and other wastes.

The data from Table 2 shows that the loss on ignition (LOI) for SSA was equal to 2.7%. The values obtained in the ash sample indicate the presence of small amounts of organic residue remaining after the SSA incineration process.

Taking into account the results presented in Table 3, it can be concluded that the content of the selected heavy metals in the SSA sample follows the sequence: Cd < Cr < Ni < Pb < Cu < Zn. For the ash sample, the copper and zinc contents are in the largest amounts. For example, the content of copper was 750 ± 3.54 mg/kg and of zinc was 2015 ± 0.09 mg/kg, respectively. The content of these elements is related to their dominance in municipal wastewater treatment plants. Werther and Ogada [55] showed that approximately 78–98% of heavy metals, such as Cd, Cr, Cu, Ni, Pb and Zn, are retained in the ash after incineration of sewage sludge. Preliminary research shows that heavy metal immobilization could be considered environmentally safe (comparable to regular construction materials). An environmental hazard is considered negligible. The leachability of heavy metals from concrete with SSA was the subject of research, among others, by Rutkowska et al. [56].

**Figure 3:**
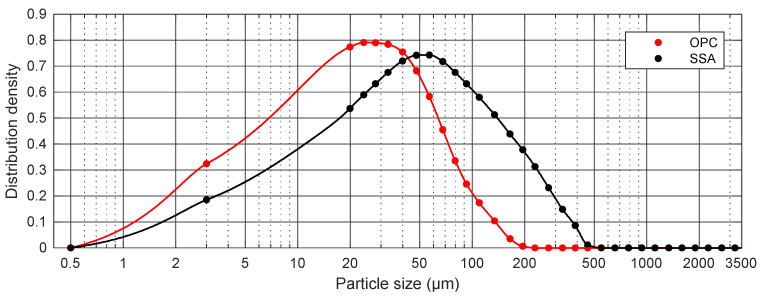
Particle size distribution curves of the tested materials (OPC and SSA).

The particle size distribution of the OPC and SSA samples obtained by the laser grain size analyzer is shown in Figure 3. The selected physical property, that is, density, D-values (D_10_, D50 and D_90_) and mean diameters (D_mean_) of the raw materials used in this study are presented in Table 4.

According to Figure 3, the OPC has a particle size mainly in the range of 18–210 μm, while the SSA particle size is mostly from 18 to 500 μm and cannot be described as strictly homogeneous. Taking into account the cumulative distribution, a relatively large fraction of the OPC particles (up to 50.4%) is less than 18 µm. For SSA particles, only about 28.7% for SSA have a diameter smaller than 18 µm. The percentage of grains of SSA in the range from 18 to 150 µm and 150 to 500 µm are respectively, 59.2% and 12.1% of the total mass.

Based on the laser grain size analysis, the particle size of the tested ash (SSA) for D_10_ is 6.6 µm, for D50 it is 39.9 µm and for D90 it is 167.75 µm (Table 4), where dv_10_, dv_50_ and dv_90_ represent the size below which 10, 50 and 90%, respectively, of the sample falls. The mean diameters (VMD) for the OPC and SSA are 27.4 µm and 66.4 µm, respectively. The specific density of SSA is 2.45 g/cm^3^ and is lower compared to the cement density (Table 4).

The results of the study presented in Figure 3 and Table 4 show that the degree of the particle size distribution of SSA is greater compared to the degree of the particle size distribution of cement.

The fineness of the tested ash (SSA)—46.8% of the total mass—does not meet the requirements of the EN-450-1 standard [51], (cat. N ≤ 40.0% mass cat. S ≤ 12.0% mass). The result shows that the use of SSA will have a negative impact on the workability of concrete and cement mixtures.

### 3.2. Heat of Hydration

**Figure 4:**
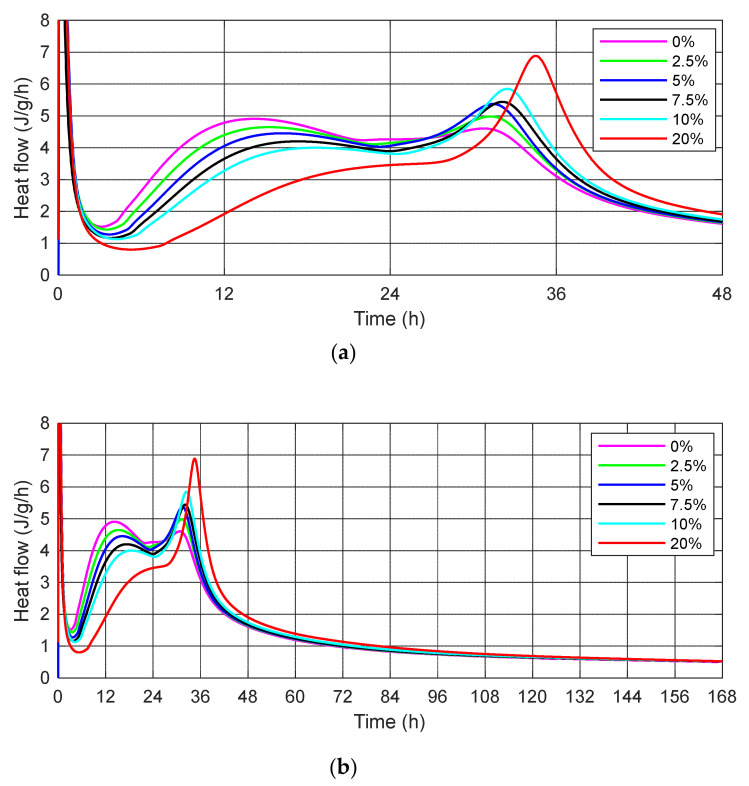
The rate of heat evolution of cement mortars (dQ/dt) without and with 2.5%, 5%, 7.5%, 10% and 20% sewage sludge ash (SSA) curing at 23 °C during (**a**) first 48 h; (**b**) 168 h.

The results of the heat generation rate for cement mortars with SSA participation in amounts of 0%, 2.5%, 5%, 7.5%, 10% and 20% of the cement mass are shown in Figure 4.

Figure 4 shows the heat flow per gram of each cement binder for 168 h (7 days) from the start of cement hydration without and with the SSA. The results obtained show that an increase in the amount of ash generates a lower value of the maximum first peak of heat flow and delays its occurrence. The curves presented in Figure 4 indicate the delay effect of the addition of SSA on the evolution of the hydration process.

In cement mortar samples with a higher SSA content, a higher heat is observed at the second peak, and therefore the appearance of a second peak can be explained by the reactivity of the SSA. Moreover, on the heat curves, when SSA is used, an extension of the acceleration period and a shift of the first heat peak maximum towards later times has been observed.

The first peak represents the dissolution of the surface of cement particles. After a short duration of this period, there was a dormant period (1–2 h) that featured a very low hydration rate. The second peak followed a dormant period, indicating SSA hydration after breaking the surface layer of the particles. Both figures (Figure 4a,b) indicate that the second peak occurs later for cement mortars with a higher SSA content.

According to Dyer et al. [37] although SSA does not seem to strongly influence the rate of hydration of Portland cement, a key feature of early hydration of cement mortars containing these materials is a very pronounced second peak in the rate of heat evolution. This peak appears to correspond to the conversion of the AFt phase (ettringite) to the AFm phase. It is also likely that the ash reacts to produce a calcium silicate hydrate (C-S-H) gel in the presence of portlandite. The results confirmed the research of Mejdia et al. [57], which showed that the alumina oxide released by SSA promotes the formation of a greater amount of AFt and AFm phases in the cement environment.

The isothermal calorimetry tests showed that the addition of SSA could prolong the induction period of cement hydration and influence the hydration rate during the acceleration period.

The values of the maximum first and second peaks of the heat evolution rate (dQ/dt) and time (t) taken to reach these values are presented in Table 5.

The data presented in Table 5 indicate that the values of the first peaks of heat flow decrease with the increasing SSA content. The time to reach the first peak of the heat generation rate is delayed for the SSA mortar compared to the control cement mortar. The maximum value of the first peak is 4.65 J/g/h for mortar with 2.5% SSA (at 15.28 h). For the cement mortar containing 20% SSA, the maximum value of the first peak was not observed on the curves of the rate of heat evolution of the cement mortar tested (Figure 4).

Taking into account the second peak of heat flow, an increase in the amount of the ash generates an increase in the second peak. For example: 4.98 J/g/h for cement mortar with 2.5% SSA (at 31.25 h) and 6.88 J/g/h for cement mortar with 20% SSA (at 34.52 h). The degree of fragmentation of the tested material compared to the particle size of OPC 42.5 R may affect the dissolution rate, including the rate of formation of chemical compounds in the structure of the hydrating cement mortars, which in turn may affect the heat of hydration.

**Figure 5:**
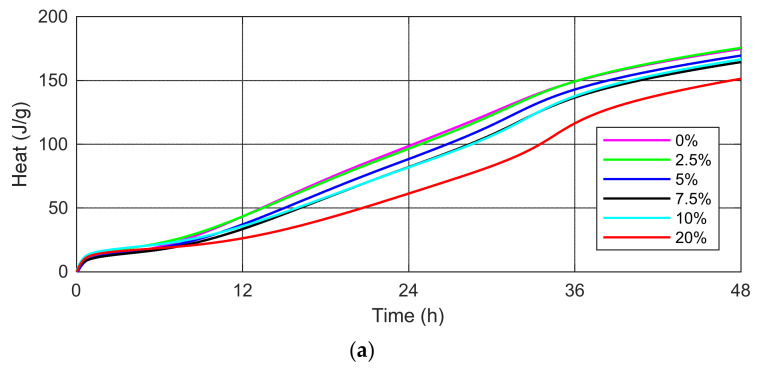

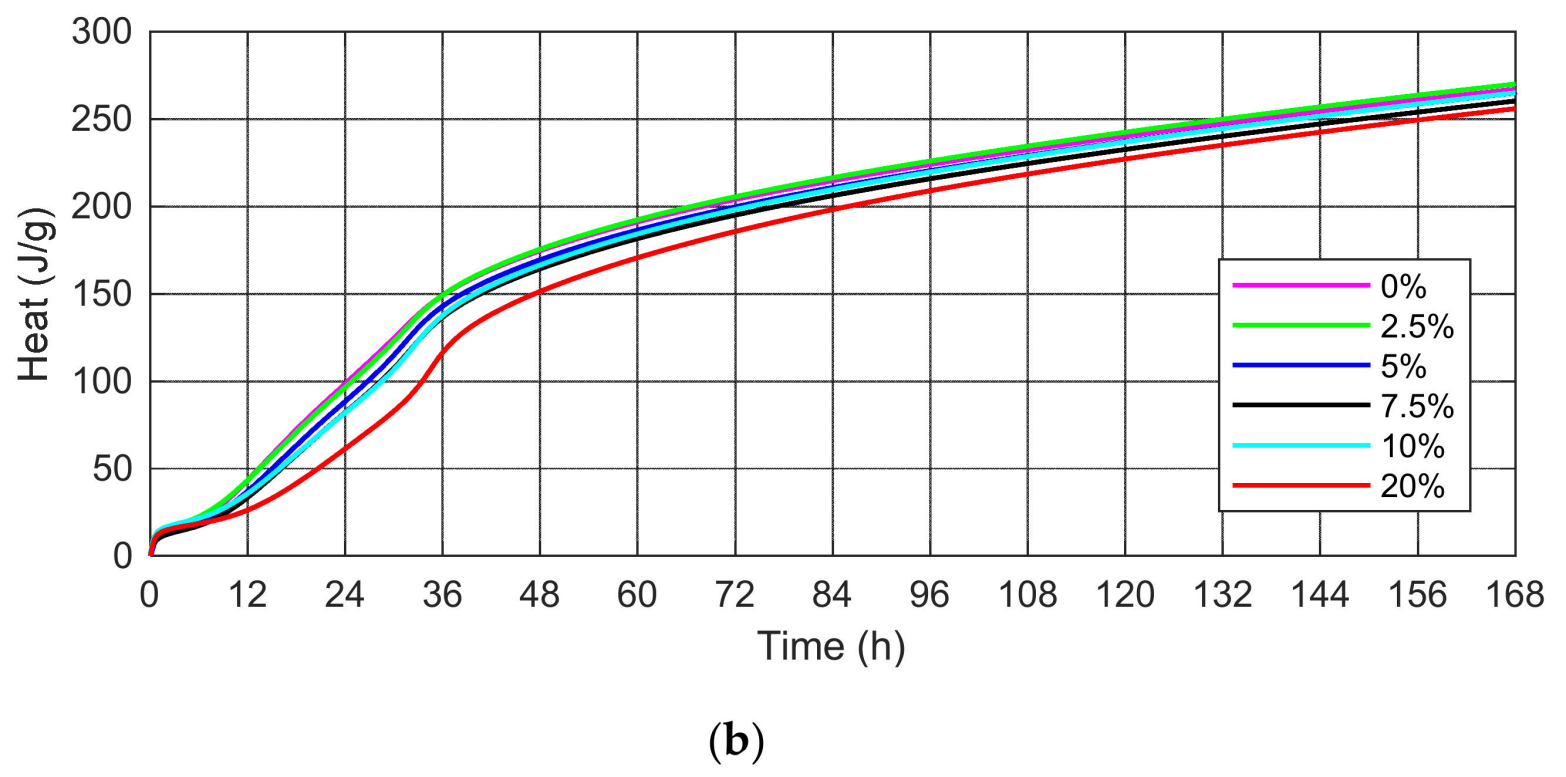
The total heat evolved for cement mortars (Q(t)) without and with 2.5%, 5%, 7.5%, 10% and 20% sewage sludge ash (SSA) cured at 23 °C during: (**a**) first 48 h; (**b**) 168 h.

The evolution of cumulative heat evolved for cement mortars without and with SSA recorded over 168 h is presented in Figure 5. The specific values of the heat released after 1, 7, 24 and 168 h are presented in Table 6.

The measured values showed that the total cumulative heat for SSA cement mortars ranges from 255.9 to 267.1 J/g after 168 h (7 days). For the control cement mortar, the heat released at 168 h is 267.1 J/g, respectively. The results presented in Table 6 suggest that a higher SSA content reduces cumulative heat after 24 and 168 h. The results of the study show that the cumulative heat rate of hydration of cement mortars after 168 h decreased with an increase in the SSA content in the samples. The total heat loss of the cement mortar with SSA is 2.12% (for 2.5%) and 37.75% (for 20%) compared to the control cement mortar after 24 h. After 168 h, the heat decrease is equal to 0.79% (for 2.5% SSA) and 4.19% (for 20% SSA) compared to the control cement mortar.

Reduction of the heat of hydration has a positive effect on the concrete hardening process (especially in the case of massive structures) due to the reduction of the risk of concrete microcracks at an early age. The tests confirmed that reducing the amount of cement in cement mortars and a proportional increase in SSA causes a decrease in the maximum heat of hydration. The rate of heat release and total heat constantly drops as the amount of SSA in cement mortars increases, which is caused by a slower rate of SSA reaction with cement.

Portland cement is an exothermic process in which the heat determines the rise in the temperature of the concrete at an early age. The maximum rate of heat evolution and the total heat are reduced due to the slower rate of the pozzolanic reaction of SSA. An increasing percentage of SSA, means less heat generation and, consequently, the temperature drops. The tested ash (SSA) is characterized by a high content of calcium and phosphorus, which reduces the rate of heat hydration. The cement hydration process is probably slower as a result of the adsorption of orthophosphate ions at the dissolution sites of the clinker phase.

**Figure 6:**
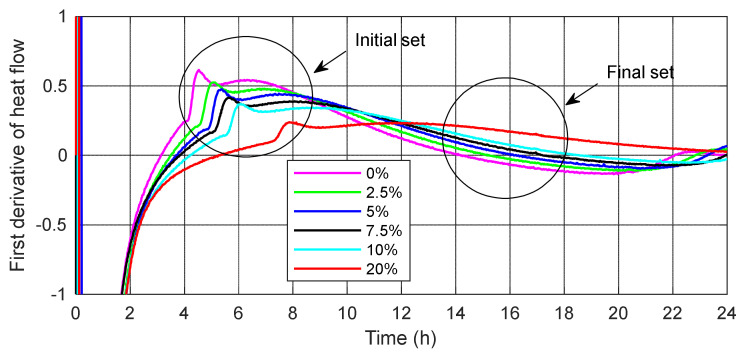
Initial and final setting time for the cement mortars without and with 2.5%, 5%, 7.5%, 10% and 20% sewage sludge ash SSA.

The next part of this study is devoted to determining the setting times of all mortars. Hu et al. [58] developed an innovative procedure to predict setting times based on calorimetry tests. The research indicated that the initial setting time of the calorimetry could be defined as the time when the first derivative of the heat evolution curve reaches its highest value. At this point, the increase in the heat flow is the fastest, and then the first derivative value decreases. The calorimetry final setting time corresponds to the first derivative equal to zero. At this point, the highest rate of heat generation is reached and after that, the rate of hydration will be reduced. The results of the initial and final calorimetry times of the mortars tested without and with SSA are shown in Figure 6 and Table 7.

The results presented in Table 7 indicate that both the initial and final setting times of the tested cement mortars are longer with increasing SSA content, which is the reason for the slower cement hydration process. In most of the cases considered, the calorimetric setting times are longer for SSA cement mortars compared to the control mortar. As expected, calorimetry setting times are the longest for cement mortars with 20% SSA due to delay of delaying hydration by adding ash. With a 20% replacement of cement with ash, the initial setting time is increased by 3.34 h compared to the control cement mortar. In turn, the final setting time is extended by approximately 10.88 h (for SSA) compared to a cement mortar without SSA. This could be explained by the slowdown of the C_3_S hydration process due to adsorption of orthophosphate ions at the site of dissolution of this phase.

Yusuf et al. [5] suggest that the setting time is influenced by the degree of fragmentation of SSA. The fineness, i.e., residue on 0.045 mm sieve of tested ash, is 46.8% (Table 4). According to Cenni et al. [12] and Vouk et al. [6] the effect of SSA on the setting time is determined by the chemical composition.

### 3.3. The Pozzolanic Activity of Sewage Sludge Ash

The pozzolanic activity of the SSA doped mortars after 28 and 90 days of curing is presented in Table 8. SAI (in %) was calculated based on the average compressive strength of the mortar sample with SSA (MPa) and the average compressive strength of the control mortar (MPa).

Partial substitution of the cement mass (25%) in the SSA resulted in a decrease in compressive strength compared to the reference sample (100% OPC 42.5 R), both at 27 and 90 days. Table 8 shows that the average pozzolanic activity of the SSA-doped mortar is 72.38% (at 28 days) and 73.3% (at 90 days). According to EN 450-1 [49], the acceptable level of pozzolanic activity is achieved when the compressive strength of 28-day-cured samples of cement—fly ash mortar constitutes 75% of the value of the control sample and after 90 days the value reaches at least 85%.

The studies presented by Fontes et al. [19], Monzo et al. [20] and Pan et al. [32] confirmed that SSA-doped mortars reached the standard value (85%) after a curing period of more than 90 days, which allows classification of this type of waste as a mineral additive with low pozzolanic activity.

### 3.4. The Compressive Strength

**Figure 7:**
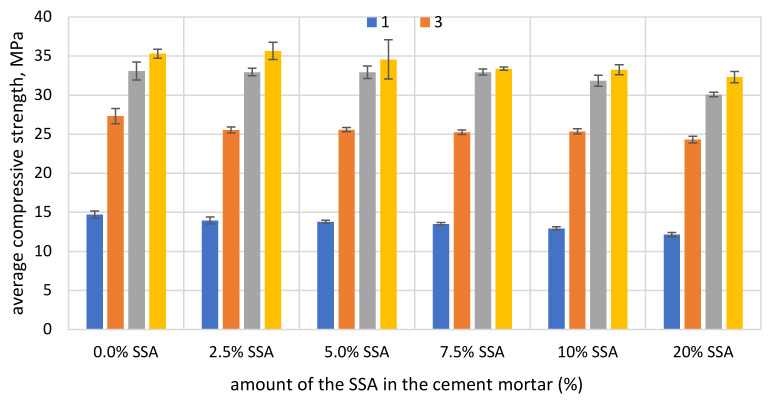
Average compressive strength of the cement mortar without and with SSA.

The compressive strength of the cement mortar samples at 1, 3, 5 and 7 days (curing at 23 °C) is presented in Figure 7. The experimental results show that at the early stage (7 days), the addition of SSA reduces the compressive strength of the mortar. The compressive strength of mortar samples decreases with increasing ash content. At 7 days curing, the highest average compressive strength, equal to 35.31 MPa, was achieved by the control sample without ash from sewage sludge. The lowest strength was 32.31 MPa for the mortar sample where 20% of the cement was replaced with SSA. In the case of the cement mortar with addition of 2.5% SSA, the average compressive strength was 35.65 MPa and was slightly higher (by 0.95%) compared to the value of the control mortar. The average compressive strength of the mortar with 5%, 7.5% 10% and 20% SSA decreases approximately by 2.1%, 5.4%, 5.9% and 8.5% compared to the control cement mortar.

The low compressive strength value at the early age (7 days) is associated with a slow and continuous reaction of ash from sewage sludge in cement mortars. The results obtained indicate that SSA, due to its chemical composition, has an impact on the cement binding hydration process. It is likely that it acts mainly as an addition with low pozzolanic activity. It may be because the SSA grains trapped a significant amount of water and a limited amount of CH was produced from cement hydration to participate in the pozzolanic reactions with SSA and provide strength to the mortar samples.

As shown in Figure 7, the strengths of all mortar samples with SSA up to 20% by weight of cement were lower, indicating a slow activation of SSA in mineral binders. Reactive silica, which is part of the total silica of SSA, probably reacts with calcium hydroxide (CH) produced from Portland cement hydration and forms calcium silicate hydrates (C-S-H gel) with a Ca/Si ratio higher than that of the C-S-H gel formed during the Portland cement hydration. Most of the durability parameters exhibited by hydrated cement paste—specifically strength—can be attributed to the C-S-H gel. Perhaps, the PO_4_^3−^ ions present in the sewage sludge ash dissolve in the liquid phase of the mineral binder, causing delays in the dissolution of C_3_S and resulting in a relatively low exothermic hydration rate of the cement particles. The test results obtained indicate that the SSA can be used as a lower activity pozzolana in cement binders. If the SSA content is added above the optimum value, this amount may not be fully involved in the chemical reaction process. It can act mainly as a mineral filler, not as a cement additive. The results suggest the need for further research to determine the function of the SSA in mineral binders.

### 3.5. The Scanning Electron Microscopy

**Figure 8:**
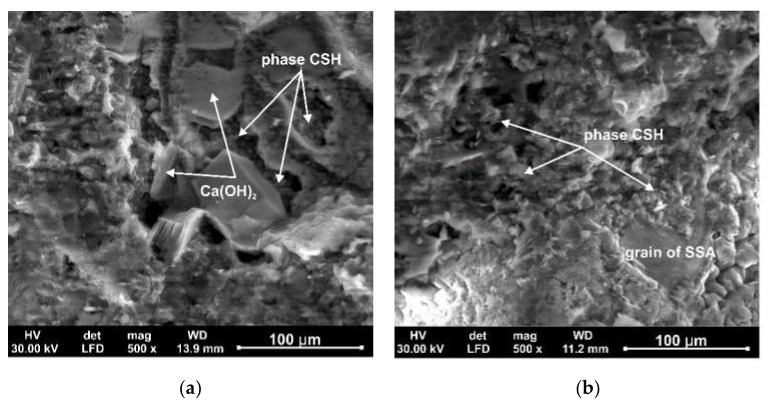
SEM microscopic image structures (500×): (**a**) OPC 42.5 R; (**b**) with sewage sludge ash after 7 days, magnification 500×.

The scanning electron microscopy (SEM) images of the structure of the tested cement mortars without and with (SSA) after 7 days of maturation are shown in Figure 8.

The microstructure of the control cement mortar presented in Figure 8a after 7 days of maturation indicates the formation of reaction products of the cement hydration process. Large crystals of Ca(OH)_2_ portlandite are visible, one of the products of the cement hydration process. The spaces of the hardened structure of the tested sample are filled with fine-crystalline C-S-H phase (hydrated calcium silicates). The C-S-H needles have grown into each other and merged into a single matrix, except at the larger openings and gaps shown in Figure 8a. The structure of the control cement mortar after 7 days of maturation includes of unreacted C_3_S (3CaO⋅SiO_2_), C_4_AF (4CaO⋅Al_2_O_3_⋅Fe_2_O_3_) and C_3_A (3CaO⋅Al_2_O_3_), phases. There are also visible unfilled pores (voids) with binding products of the cement hydration process. The presented microstructure (Figure 8b) of the cement mortar with SSA, indicates that the products of the cement hydration process surround the grains of the tested material. The observed forms are probably a fine-crystalline form of hydrated calcium silicates, which is formed as a result of the dissolution of the tested material in the environment of hydrating cement. A small amount of portlandite is visible in the pores of the mineral binders.

### 3.6. X-ray Diffraction (XRD) Analysis

**Figure 9:**
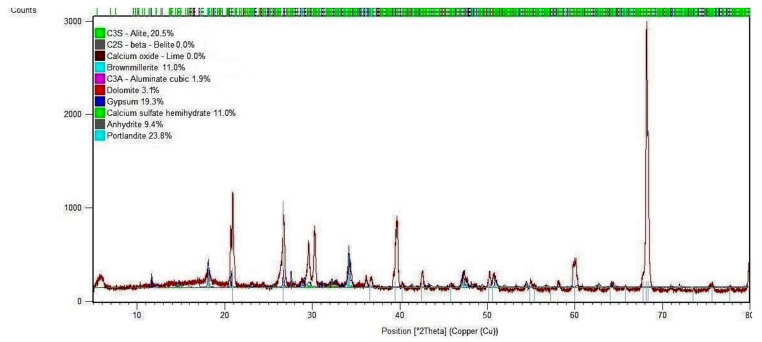
X-ray diffraction pattern of control mortar sample (OPC 42.5 R) after 7 days.

**Figure 10:**
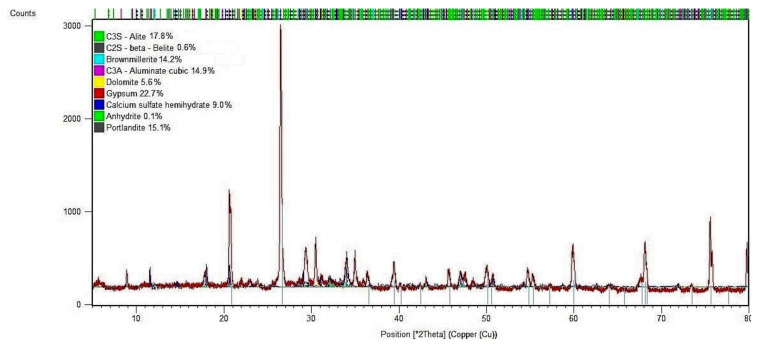
X-ray diffraction pattern of mortar sample with 20% SSA after 7 days.

The results of the mineralogical composition (in the form of a diffraction pattern) of mortar samples without and with 20% SSA at the age of 7 days are shown in Figure 9 and Figure 10.

The result of the XRD analysis of the control sample (OPC only) shows several major diffraction peaks that can be used to assess cement hydration. Generally, the early hydration of Portland cement is controlled by the dissolution of C_3_A, C_3_S and gypsum. In turn, the dissolution and consumption of C_3_A and gypsum is related to the formation of ettringite.

The peak corresponding to the cement hydration product CH (Ca(OH)_2_) appears at 17.8° position, and the peaks corresponding to C_3_S (3CaO·SiO_2_) and C_2_S (2CaO·SiO_2_), are located respectively at 27.6°, 31.1°, 34.4°, 41.3°, 51.7° and 56.6°, are discussed in this study. Among them, the most prominent peaks are seen, which appear at 32.3°, 32.7° and 34.5°, according to the amount of unhydrated cement clinker.

The main crystalline phases visible in Figure 9 in the mortar sample without SSA cured for 7 days are calcium-silicate hydrate (C-S-H gel)—11.0%, portlandite (Ca(OH)_2_—23.8%, two forms of gypsum (unhydrated and hydrated)—19.3% and 9.3% and dolomite (CaCO_3_)—3.1% in the total sample. The structure of the tested sample contains unreacted clinker phases in the form of: tricalcium silicate (C_3_S)—20.5%, tetracalcium aluminoferrite (C_3_AF)—11.0% and tricalcium aluminate (C_3_A)—1.9%. One of the peaks (in the 68° position) indicates the dominance of calcium hydroxide in relation to other compounds present in the control mortar.

The analysis of the crystalline composition (XRD analysis) and the morphological composition (SEM analysis) of cement mortar with the addition of 20% SSA indicates slight differences compared to the control mortar sample (0% SSA) after 7 days of curing, which underlines the possible influence of SSA on the heat hydration process.

As shown in Figure 10, hydration products were primarily composed of the phase C-S-H—9.0%, portlandite—15.1%, two form CaSO_4_ (unhydrated and hydrated)—22.7% and 0.1% and CaCO_3_—5.6% in the total sample.

The XRD test showed that the structure of the tested sample contains unreacted clinker phases in the form of the following: C_3_S—17.8%, C_3_AF—14.2% and C_3_A—14.9%. The delay in cement hydration, caused by the use of SSA, can be explained by the presence of orthophosphate ions (PO_4_^3−^), which may inhibit crystal growth in the tested samples. The adsorption of phosphates ions at the reactive sites of the mineral binder probably inhibits the dissolution rate of the clinker phases. The performed XRD tests did not confirm the formation of chemical compounds containing phosphorus ions in the mineral sample with the addition of 20% SSA, in such a short time (7 days) of the hydration process. In the tested sample, there was an increase in CaSO_4_ and a decrease in the phase content of C-S-H and Ca(OH)_2_ compared to the control sample.

XRD analysis of a cement mortar substituted with 20% SSA showed that the intensity of the peaks is essentially similar to that of the Portland mortar sample, but the height of the diffraction peaks is clearly reduced.

The results of the diffractograms are in good agreement with our previous results obtained with the hydration and strength tests.

## 4. Conclusions

Based on the results of the selected properties of SSA and the thermal parameters of the cement mortars with SSA, the following conclusions can be drawn:

1 The main components of the SSA samples are SiO_2_, CaO and P_2_O_5_. However, other oxides, such as Al_2_O_3_, Fe_2_O_3_, MgO, K_2_O and adsorbed SO_3,_ are also present, but in a smaller amount. The phosphorus content is particularly significant compared to other components due to the characteristic of sewage sludge being incinerated and the influence of the mentioned phosphorus species on the final mortar-based products. The sum of silica, aluminum and iron oxides does not meet the requirements of the standards (ASTM C618 in the USA and EN 450 in Europe) dedicated to fly ash from conventional coal combustion.
2 The use of this type of ash in concrete technology should be preceded by obtaining European technical approvals. According to EN 450-1 there is a possibility of obtaining fly ash from the co-combustion of sewage sludge with coal under proper conditions. However, it cannot be done within the wastewater treatment plant, where the fluidized bed furnace is considered BAT (best available technology).
3 The substitution of cement by SSA delays the evolution of the hydration process compared to the control cement mortar. SSA samples are characterized by a high content of calcium and phosphorus, which reduces the rate of heat hydration. The increasing amount of ash causes a lower value of the maximum first peak of heat flow and delays its occurrence.
4 The impact on the binding time of slurries, apart from the water demand, is mainly the phosphorus oxide content in the binder. A small change in the mineral supplement rich in phosphorus can cause a sharp decline in the initiation or elongation of hydration, creating a critical interval that occurs at a dosage of 5–10% ash.
5 The initial and final setting times of the tested mortars are longer with increasing SSA content, which is caused by a slower rate of SSA pozzolanic reaction and slower development of the microstructure. The heavy metals in SSA probably affected the hydration of the cement and therefore the initial and final setting time of the mortars.
6 The pozzolanic activity of SSA does not meet the requirements of the standard EN 450-1 after 28 days (≥75%) and 90 days (≥85%) of curing. The addition of SSA reduces the compressive strength of mortar samples in the early stages of development.
7 The higher SSA content reduces the cumulative heat of hydration, thus favorably affecting the hardening process of massive structures due to the reduction of the risk of concrete microcracks at an early age.

The procedure using isothermal calorimetry is extremely precise and useful for determining the setting time of cementitious materials. Fly ash from thermal treatment of sewage sludge is a unique material with the potential to be used in construction materials. These aspects of the circular economy concept were mainly tested by utilization of SSA in cementitious materials as a cement substitute. The preliminary investigations of physicochemical properties of SSA and the isothermal calorimetry tests of the cement mortars enhanced by SSA prove that there are possibilities to recycle this kind of ash in building materials. The confirmation of this fact requires further research depending on the type and combustion conditions of sewage sludge.

## Figures and Tables

**Table 1:** Composition of the cement mortars without and with sewage sludge ash (SSA).

SSA Substitution Rate (%)	Components of Cement Mortar, kg/m^3^				w/b (-)
	OPC 42.5 R	SSA	Sand	Water	
0.00	390.00	0.00	1085.0	214.0	0.55
2.50	380.25	9.75	1085.0	214.0	0.55
5.00	370.50	19.50	1085.0	214.0	0.55
7.50	360.75	29.25	1085.0	214.0	0.55
10.00	351.00	39.00	1085.0	214.0	0.55
20.00	312.00	78.00	1085.0	214.0	0.55

**Table 2:** Chemical composition of the tested raw materials (by % weight) determined via X-ray EDS.

Determined Species and Parameters	OPC 42.5 R	Sewage Sludge Ash (SSA)	Admissible Content acc. to EN 450-1 [51]
SiO_2_ (%)	21.2	18.9	≤25% mass
Fe_2_O_3_ (%)	2.2	13.8	-
Al_2_O_3_ (%)	5.0	9.6	-
The sum of oxide content (SiO_2_ + Fe_2_O_3_ + Al_2_O_3_)	28.4	42.3	≥70% mass
CaO (%)	64.1	18.2	≤10% mass ^1^
CaO_free_ (%)	-	-	-
MgO (%)	2.5	4.4	≤4.0% mass
SO_3_ (%)	3.0	2.1	≤3.0% mass
Na_2_O (%)	0.13	1.1	-
K_2_O (%)	0.92	2.2	-
Na_2_O_eq_ (Na_2_O + 0.658K_2_O)	0.74	2.6	≤5.0% mass
P_2_O_5_ (%)	-	25.1	≤5.0 mg/kg
TiO_2_ (%)	-	1.5	-
Chloride content (%)	0.03	-	≤0.10% mass
Loss on ignition LOI (%)	3.1	2.7	Cat.: A ≤ 5%; B 2 ÷ 7% C 4 ÷ 9% mass

^1^ if the total content of CaO in the fly ash has lower than 10% mass of the requirement for reactive CaO it is deemed to be fulfilled

**Table 3:** The general contents of selected heavy metals in SSA sample.

Type of Sample	Type of Determined Element, (mg/kg) Dry Weight					
	Cadmium (Cd)	Copper (Cu)	Nickel (Ni)	Lead (Pb)	Zinc (Zn)	Chrome (Cr)
SSA	3.3 ± 1.32	750 ± 3.54	68.3 ± 0.05	88.7 ± 2.01	2015 ± 0.09	54.2 ± 1.03

**Table 4:** Particle size (D_10_, D_50_, D_90_, D_mean_), density and fineness of OPC and SSA.

Samples	OPC	SSA
D_10_ (µm)	3.97	6.59
D_50_ (µm)	17.86	39.86
D_90_ (µm)	62.04	167.75
VMD (D_mean)_ (µm)	27.41	66.42
Specific density (g/cm^3^)	3.05	2.45
Fineness *, %	19.40	46.80

* residue on 0.045 mm sieve according to EN 451-2 [45].

**Table 5:** The characteristic peak values of heat flow for cement mortars without and with SSA.

Peak and Time Value of the Heat Flow			Content of the Sewage Sludge Ash in the Cement Mortar					
			0%	2.5%	5.0%	7.5%	10%	20%
first	dQ/dt	(J/g/h)	4.91	4.65	4.45	4.20	4.00	-
	t	(h)	14.20	15.28	16.25	17.28	18.55	-
second	dQ/dt	(J/g/h)	4.60	4.98	5.37	5.44	5.85	6.88
	t	(h)	30.77	31.25	31.45	32.13	32.48	34.52

**Table 6:** Characteristic values of the hydration heat (J/g) evolved after hours for the cement mortars.

Mortar Type	Time (h)						
	1	7	24	48	72	120	168
0.0% SSA (J/g)	9.91	22.58	98.54	174.6	203.99	240.24	267.1
2.5% SSA (J/g)	13.31	24.97	96.45	175.5	205.49	242.49	265.0
5.0% SSA (J/g)	10.36	20.78	88.40	169.4	199.73	237.18	264.9
7.5% SSA (J/g)	10.03	19.16	82.10	164.4	195.12	232.69	260.3
10% SSA (J/g)	14.14	23.17	81.87	160.5	198.18	236.87	258.0
20% SSA (J/g)	12.51	19.44	61.34	151.3	185.58	227.03	255.9

**Table 7:** The determination of calorimetry set times for cement mortars with SSA.

Setting Time (h)	Content of Sewage Sludge Ash in the Cement Mortar					
	0%	2.5%	5.0%	7.5%	10%	20%
initial	4.53	5.12	5.38	5.63	6.07	7.87
final	14.22	15.17	16.22	17.18	18.57	25.10

**Table 8:** Strength activity indexes (SAI) of sewage sludge ash sample obtained with cement.

Days	Mortar Type	Average Compressive Strength, MPa	SD *, MPa	Strength Activity Index (SAI)
28	100% OPC 42.5 R	56.49	3.56	72.38%
28	75% OPC + 25% SSA	40.89	1.42	
90	100% OPC 42.5 R	59.48	2.67	73.30%
90	75% OPC + 25% SSA	43.60	1.38	

* Standard deviation.

## Data Availability

The data supporting reported results are not stored in any publicly archived datasets. Readers can contact the corresponding author for any further clarification of the results obtained.
